# Perceptions of the Malaysian general public on community pharmacy-based weight management services

**DOI:** 10.1186/s40545-018-0146-x

**Published:** 2018-08-08

**Authors:** Rohit Kumar Verma, Thomas Paraidathathu, Nur Akmar Taha, Wei Wen Chong

**Affiliations:** 1 Department of Pharmacy Practice, School of Pharmacy, International Medical University, 57000 Kuala Lumpur, Malaysia; 2 Faculty of Health and Medical Sciences, Taylor’s University, 47500 Subang Jaya, Malaysia; 3 Faculty of Pharmacy, Cyberjaya University College of Medical Sciences, Edusphere, Persiaran Bestari, 63200 Cyberjaya, Selangor Malaysia; 4 Faculty of Pharmacy, Universiti Kebangsaan Malaysia, 50300 Kuala Lumpur, Malaysia

**Keywords:** Community pharmacists, General public perception, Malaysia, Weight management services

## Abstract

**Background:**

Obesity is now widely regarded as a main contributor to poor health. Involvement of community pharmacists can be a valuable tool in obesity management. However, there is still a lack of data in Malaysia on the potential involvement of and opportunities for community pharmacists in providing weight management services. Thus, it is essential to investigate the perceptions of the general public on weight management services in the community pharmacy setting. To evaluate the general public’s perceptions on weight management services by community pharmacists in terms of perceived availability, utilization and factors influencing acceptability of services.

**Methods:**

A descriptive, cross sectional-survey was conducted using a self-administered questionnaire comprising of sections that focused on public preferences and options on weight management approaches, perceived availability of extended services and resources provided by community pharmacists in relation to weight management, utilization of these services and resources, and factors influencing acceptability of weight management services provided by community pharmacists. The questionnaires were distributed to the general public aged 18 years and above in Klang Valley, Malaysia.

**Results:**

A total of 730 respondents with a median age of 31 years participated in this study. Majority of respondents ranked dieticians as their preferred first line of consultation, with only about a quarter of respondents ranking community pharmacists as their preferred first or second line of consultation. Although more than half show of the study respondents perceived that community pharmacies they had visited offered services for measuring weight, height, blood pressure, blood glucose and blood cholesterol, fewer perceived that community pharmacies provided advice on physical activity and healthy eating to achieve weight loss. Additionally, majority of the respondents indicated that they had not utilized these services. However, most respondents perceived that community pharmacists should provide weight management services. The main factors influencing acceptability show of services included training of pharmacists, payment, waiting time and the issue of privacy.

**Conclusion:**

The findings of this study demonstrated that the majority of respondents were in support of weight management services in community pharmacy; however only a low percentage reported utilizing these services. Factors influencing acceptability of services included payment, waiting time and the issue of privacy. With adequate training among pharmacists and increased awareness of services among the public, community pharmacists could play a larger and important role in addressing the issue of obesity in Malaysia.

## Background

In recent years, obesity has become a serious health problem that often leads to multiple health risks. According to the World Health Organization (WHO), any person with body mass index (BMI) of 25–29.9 kg/m^2^ is categorized as overweight and more than 30 kg/m^2^ as obese [1]. Obesity is recognised as a major determinant of non-communicable diseases such as diabetes, heart diseases, respiratory disorders, cancers, gallbladder diseases, post-operative morbidity and musculoskeletal disorders such as osteoarthritis [2–4].

Obesity has become a leading health issue in many countries in Southeast Asia including Malaysia [5]. Malaysia has recently been ranked second highest in East and Southeast Asia in terms of the population being overweight. The Third National Health and Morbidity Survey (NHMS III) in 2006 conducted among 33, 055 adults found 29.1% were overweight and 14.0% obese [6]. The following NMHS conducted in 2011 and 2015 showed increases in the rates of those overweight and obese [7]. Like any other chronic disease, overweight and obesity hugely impact direct and indirect economic consequences on the healthcare system [8, 9]. Hence it is worthwhile to seek a national strategy for tackling these problems [10].

As in most chronic conditions, the management of obesity requires a multidisciplinary team-based approach involving various healthcare professionals such as physicians, nurses, pharmacists, physiotherapists and dieticians. In many countries, community pharmacists are also involved in addressing this problem. The role of community pharmacists has expanded to include disease prevention and supporting public health initiatives [11]. Weight management can also be considered as an extended service offered by community pharmacists with a potential to impact public health [12, 13] . Moreover, community pharmacists are easily accessible and possess wide knowledge on medicines, diseases and lifestyle modification. Pharmacists can contribute to weight management in multiple ways including screening [14], face-to-face behavioral therapy sessions [15], pharmacist-managed weight management clinics [16], and dosage adjustment in obese patients [17], among others.

In a country with an adequate number of pharmacists, it is worth utilizing the expertise and skills available among community pharmacists to enhance health outcomes and reduce long-term effects associated with obesity. Over the last decade, the community pharmacy profession in Malaysia has been undergoing a paradigm shift in the focus of its practice. Many community pharmacies supply over-the-counter weight loss products and often even conduct weight loss programmes as a part of their extended service programs [18]. In this view, it is also important to evaluate the perceptions of the Malaysian general public to whom community pharmacy-based weight management services would be targeted. Therefore, the aims of the current study were to evaluate the general public’s opinion on preferences and options on weight management interventions, and their perceptions on weight management services by community pharmacists in terms of availability, utilization and factors influencing acceptability of services.

## Methods

### Study design

A cross-sectional survey was conducted to gather the views of the general public in Klang Valley, Malaysia on weight management services in community pharmacy. The survey was conducted from September 2016 to January 2017.

A significant sample size of 384 respondents was calculated to meet a statistical defined margin of 5% error and 50% response distribution using Krejcie and Morgan sample size estimation method [19]. Convenience sampling was used to recruit study respondents. Respondents were included if they were over 18 years of age, and had visited community pharmacies previously for health-related matters. Respondents who had difficulty in understanding English or Malay languages were excluded from the study.

### Survey instrument

A self-administered questionnaire was designed after a thorough review of the relevant literature related to obesity and community pharmacy services. The questionnaire consisted of 56 items in nine sections related to respondents’ perceptions on methods to achieve weight reduction; preferences of weight management options; weight management services provided by community pharmacies; weight management products and resources available at community pharmacies; utilization of weight management services at community pharmacies; advice and referrals related to weight management received at community pharmacies; purchase of products related to weight management in community pharmacies; barriers in obtaining community pharmacy weight management services; and sociodemographic characteristics of respondents. Excluding questions for demographic data, responses were recorded either through a five-point Likert scale (1 = strongly disagree, 2 = disagree, 3 = not sure, 4 = agree and 5 = strongly agree) or selection of options (Yes, No, Not sure). In the section on preferences of weight management options, the respondents were asked to rank their preferences in getting consultation help for weight reduction from professionals such as dieticians, doctors, pharmacists, nurses, fitness instructors or staff at slimming centers. The questionnaire was available both in the English and the Malay language.

The questionnaire was evaluated for content and face validity by experts (3 community pharmacists and 2 academic pharmacists) who had ten or more years of experience in the field of community pharmacy practice research in Malaysia. In addition, the questionnaire was pilot-tested with 30 subjects. The overall Cronbach’s alpha was 0.764, and hence, the instrument was considered to have adequate reliability.

### Data collection

Data were collected by face-to-face distribution of the questionnaires. The questionnaires were distributed to respondents who had visited selected community pharmacies, weight management training centers, slimming centers and fitness centers. The information sheet about the background to the study and its objectives were also included in the set of questionnaires. Participation in the study was voluntary, and written consent was obtained from the respondents prior to data collection.

### Ethics

Ethical approval for this study was obtained from the Ethics Committee of UKM Medical Centre, Kuala Lumpur, Malaysia (NF-016-14).

### Data analysis

Data were analysed using Statistical Package for the Social Sciences (SPSS) version 22.0 software. Descriptive statistics were used to summarize participants’ responses. The association between participants’ response to the question on whether community pharmacists should provide weight management services and their demographic variables were investigated for statistical significance using Chi-squared and point-biserial correlation tests. Statistical significance was set at *P* < 0.05.

## Results

A total number of 730 respondents with a median age of 31 years participated in this study. Overall, 54.2% of the respondents were female (*n* = 396) and 44.9% were of Malay ethnicity (*n* = 328). About half of the respondents (*n* = 316; 43.3%) had a Bachelor’s degree as their highest level of education. The median monthly income of the respondents was 2000 Malaysian Ringgit (approximately USD513.62). When asked to describe body image, majority of respondents perceived themselves as overweight (*n* = 461, 63.2%), followed by obese (*n* = 155, 21.2%). The actual body mass index (BMI) of respondents (measured and calculated during the study by using Sokano Digital Weighing Machine) also showed similar distribution, with 60.8% of respondents characterized as overweight and 27% as obese. Further details about the social, demographic and economic characteristics of the respondents are shown in Table 1.

**Table 1:** Sociodemographic characteristics of respondents (*n* = 730)

		*n*	%
Age (years) (Mean ± SD; range)		33.46 ± 11.00, 18–84 years	
Gender			
Male		334	45.8
Female		396	54.2
Ethnicity			
Malay		328	44.9
Chinese		235	32.2
Indian		162	22.2
Others		5	0.7
Highest level of education			
No formal education		7	1.0
Primary education		21	2.9
Secondary education		54	7.4
Diploma		269	36.8
Bachelor’s degree		316	43.3
Master degree		45	6.2
Doctorate degree		3	0.4
Others		15	2.1
Monthly income (RM) (Mean ± SD; range)		3161 ± 1869, 500–20,000	
Body image described by participant-self described			
Underweight (< 18.50 Kg/m^2^)		4	0.50
Normal weight (18.50–24.99 Kg/m^2^)		110	15.1
Overweight (≥25 Kg/m^2^)		461	63.2
Obese (≥30 Kg/m^2^)		155	21.2
Actual body mass index (BMI)-Measured			
Underweight (< 18.50 Kg/m^2^)		2	0.3
Normal weight (18.50–24.99 Kg/m^2^)		84	11.4
Overweight (≥25 Kg/m^2^)		446	60.8
Obese (≥30 Kg/m^2^)		198	27

Total number may not match *n* = 730 due to missing data

### Preferences of the public in weight management

The respondents were asked to rank their preferences in getting consultation help for weight reduction from professionals such as dieticians, doctors, pharmacists, nurses, fitness instructors or staff at commercial slimming centers. Most respondents (*n* = 275; 37.7%) ranked dieticians as their preferred first line of consultation, followed by fitness instructors (29.9%) and doctors (13%) (Table 2). Only 26.6% of respondents ranked pharmacists as their preferred first or second line of consultation.

**Table 2:** Preferences as ranked by respondents in getting consultation help for weight reduction (n = 730)

Rank	Frequency (%)					
	Dieticians	Doctors	Pharmacists	Nurses	Fitness Instructors	Slimming Center Staff
1	275 (37.7)	95 (13)	62 (8.5)	2 (0.3)	218 (29.9)	52 (7.1)
2	178 (24.4)	142 (19.5)	139 (19.0)	21 (2.9)	171 (23.4)	113 (15.5)
3	118 (16.2)	250 (34.2)	147 (20.1)	30 (4.1)	99 (13.6)	112 (15.3)
4	93 (12.7)	140 (19.2)	174 (23.8)	120 (16.4)	90 (12.3)	119 (16.3)
5	53 (7.3)	83 (11.4)	194 (26.6)	117 (16.0)	123 (16.8)	144 (19.7)
6	12 (1.6)	18 (2.5)	13 (1.8)	439 (60.1)	28 (3.8)	189 (25.9)

Note: Rank (1 – Most preferred and 6 – Least preferred)

In addition, respondents were asked their opinion about the effectiveness of various weight loss measures (Table 3). The weight reduction measures most strongly agreed or agreed by respondents to be effective were exercise (*n* = 677, 92.8%) and counseling by dieticians (*n* = 598, 81.9%). About three quarters of the respondents also believed that effective treatment of obesity requires teamwork between healthcare professionals (*n* = 544, 74.5%), and between health professionals and the individual (*n* = 595, 81.5%).

**Table 3:** Opinions about effectiveness of various weight loss measures among respondents (*n* = 730)

Which of the following do you think can achieve weight reduction?	Strongly disagree	Disagree	Not sure	Agree	Strongly agree
	*n* (%)	*n* (%)	*n* (%)	*n* (%)	*n* (%)
*Measures*					
Exercise training	2 (0.3)	8 (1.1)	43 (5.9)	256 (35.1)	421 (57.7)
Counseling by dieticians	4 (0.5)	35 (4.8)	93 (12.7)	421 (57.7)	177 (24.2)
Medications	83 (11.4)	118 (16.2)	175 (24.0)	219 (30.0)	134 (18.4)
Dietary supplements	74 (10.1)	112 (15.3)	127 (17.4)	303 (41.5)	114 (15.6)
Weight loss surgery	247 (33.8)	152 (20.8)	121 (16.6)	189 (25.9)	21 (2.9)
*Effective treatment of obesity needs…*					
More than one of the methods above	14 (1.9)	53 (7.3)	165 (22.6)	358 (49.0)	140 (19.2)
Teamwork among health professionals	6 (0.8)	17 (2.3)	163 (22.3)	365 (50.0)	179 (24.5)
Teamwork between health professionals and the individual	3 (0.4)	5 (0.7)	127 (17.4)	294 (40.3)	301 (41.2)

### Perceived availability of weight management services and products in community pharmacies

Majority of the study respondents perceived that community pharmacies they had visited offered services for measuring health indicators such as blood pressure (*n* = 591, 81%), blood glucose (*n* = 581, 79.6%) and blood cholesterol (*n* = 532, 72.9%) (Table 4). Fewer respondents perceived the availability of more direct weight management services such as measurement of BMI (*n* = 337, 46.2%), waist/hip circumference (*n* = 261, 35.8%) and body fat percentage (*n* = 250, 34.2%). In addition, slightly less than half of the respondents felt that community pharmacies provided advice on physical activity (*n* = 353, 48.4%) and healthy eating (*n* = 337, 46.2%), or referrals to other healthcare professionals for weight management (*n* = 189, 25.9%).

**Table 4:** Perceived availability and utilization of weight management services and products among respondents (*n* = 730)

Weight management services and products	Perceived availability of services and products (‘Are the following services or products available at the community pharmacy that you go to?’)			Utilization of services and products obtained (‘Have you ever used the following services or bought the following products at the community pharmacy that you go to?’)		
	Yes *n* (%)	No *n* (%)	Not sure *n* (%)	Yes *n* (%)	No *n* (%)	Not sure *n* (%)
*Pharmacy services*						
Measurement of weight	409 (56.0)	276 (37.8)	45 (6.2)	234 (32.1)	396 (54.2)	100 (13.7)
Measurement of height	367 (50.3)	313 (42.9)	50 (6.8)	194 (26.6)	453 (62.1)	81 (11.1)
Calculation of body mass index	337 (46.2)	337 (46.2)	56 (7.7)	157 (21.5)	494 (67.7)	79 (10.8)
Measurement of waist/hip circumference	261 (35.8)	401 (54.9)	68 (9.3)	118 (16.2)	560 (76.7)	52 (7.1)
Measurement of body fat percentage	250 (34.2)	395 (54.1)	83 (11.4)	89 (12.2)	586 (80.3)	55 (7.5)
Measurement of blood pressure	591 (81.0)	127 (17.4)	12 (1.6)	260 (35.6)	306 (41.9)	164 (22.5)
Measurement of blood glucose	581 (79.6)	133 (18.2)	16 (2.2)	234 (32.1)	338 (46.3)	158 (21.6)
Measurement of blood cholesterol	532 (72.9)	166 (22.7)	32 (4.4)	194 (26.6)	394 (54.0)	141 (19.3)
Advice on physical activity to achieve weight loss	353 (48.4)	312 (42.7)	64 (8.8)	211 (28.9)	402 (55.1)	117 (16.0)
Advice on healthy eating to achieve weight loss	337 (46.2)	324 (44.4)	68 (9.3)	206 (28.2)	429 (58.8)	95 (13.0)
Referral to doctors and/or dieticians for weight loss	189 (25.9)	439 (60.1)	102 (14.0)	43 (5.9)	645 (88.4)	42 (5.8)
Referral to slimming centers for weight loss	141 (19.3)	477 (65.3)	109 (14.9)	35 (4.8)	653 (89.5)	41 (5.6)
Referral to other programs	25 (3.4)	479 (65.6)	85 (11.6)	9 (1.2)	505 (69.2)	99 (13.6)
*Weight management products and resources*						
Supplements (e.g. powdered drinks, herbal products and other non-drug products)	553 (75.8)	151 (20.7)	26 (3.6)	181 (24.8)	385 (52.7)	164 (22.5)
Creams	323 (44.2)	270 (37.0)	137 (18.8)	55 (7.5)	648 (88.8)	27 (3.7)
Oral medications	388 (53.2)	238 (32.6)	104 (14.2)	76 (10.4)	553 (75.8)	100 (13.7)
Information materials (e.g. brochures, leaflets, popular diet books and video CDs)	283 (38.8)	308 (42.2)	139 (19.0)	80 (11.0)	589 (80.7)	61 (8.4)

In terms of weight management products, more than half of the respondents reported that community pharmacies had supplements (such as powdered drinks, herbal products, and other non-drug products) (*n* = 553, 75.8%) and oral medications (*n* = 388, 53.2%) for reducing weight (Table 4).

### Utilization of weight management services and products in community pharmacies

Majority of the respondents reported that they had not used the services available at the community pharmacies (Table 4). This included seeking the pharmacist’s advice, whereby the majority of respondents reported that they had not sought a pharmacist’s advice regarding healthy eating (*n* = 429, 58.8%) or physical activity (*n* = 402, 55.1%) in the past. In terms of weight loss products, only 24.8 and 10.4% of respondents indicated that they have obtained supplements and oral medications respectively at the community pharmacies they visited (Table 4).

### Factors affecting acceptability of community pharmacy-based weight management services

Majority of the respondents (*n* = 470, 64.4%) perceived that community pharmacists should provide weight management services, and 40.7% (*n* = 297) perceived that community pharmacists were trained to manage overweight and obese patients (Table 5). Majority of them also preferred the weight management services to be provided in a private area (*n* = 493, 67.5%), without any additional cost (*n* = 398, 54.5%) or long waiting time (*n* = 337, 46.2%) to get the service.

**Table 5:** Responses regarding acceptability of community pharmacy weight management services

Respondents’ opinions about acceptability of community pharmacy weight management services	Yes	No	Don’t know
	*n* (%)	*n* (%)	*n* (%)
Do you think community pharmacists are trained to manage overweight and obese patients?	297 (40.7)	235 (32.2)	197 (27.0)
In your opinion, should community pharmacists provide weight management services?	470 (64.4)	164 (22.5)	96 (13.2)
Do you think people will use weight management services by community pharmacists?	424 (58.1)	167 (22.9)	139 (19.0)
Do you think community pharmacists have time to provide weight management services?	263 (36.0)	268 (36.7)	198 (27.1)
Do you think customers should pay for weight management services in a pharmacy?	225 (30.8)	398 (54.5)	106 (14.5)
Would you wait 30 min to get weight management services from a community pharmacist?	272 (37.3)	337 (46.2)	120 (16.4)
Would you want weight management services to be done privately (e.g. in a separate room)?	493 (67.5)	169 (23.2)	68 (9.3)

### Comparison between demographic characteristics and acceptability of community pharmacy-based weight management services

The chi-square analysis revealed that gender, ethnicity, educational level and self-perceived body image were significantly associated with acceptability of community pharmacy-based weight management services (*p* < 0.05) (Table 6). Females, respondents from Indian/other ethnicities, lower educational level and who perceived themselves as overweight and obese patients were more likely to agree that community pharmacists should provide weight management services (Table 6).

**Table 6:** Comparison between demographic characteristics and consumer beliefs about community pharmacist weight management services

Study characteristics	“In your opinion, should community pharmacists provide weight management services?”		χ2 or Fisher Exact	*p*-value
	Yes	No/ don’t know		
Gender				
Male	197 (59.0%)	137 (41.0%)	7.834	0.005**
Female	273 (68.9%)	123 (31.1%)		
Ethnicity				
Malay	194 (59.1%)	134 (40.9%)	23.763	0.000***
Chinese	143 (60.9%)	92 (39.1%)		
Indian	128 (79.0%)	34 (21.0%)		
Others	5 (100.0%)	0 (0.0%)		
Educational level				
No formal/ primary	21 (75.0%)	7 (25.0%)	14.115	0.015*
Secondary education	43 (79.6%)	11 (20.4%)		
Diploma	182 (67.7%)	87 (32.3%)		
Bachelor’s degree	183 (57.9%)	133 (42.1%)		
Masters/ PhD	32 (66.7%)	16 (33.3%)		
Others	9 (60.0%)	4 (40.0%)		
Self-perceived body image				
Underweight/ normal weight	62 (54.4%)	52 (45.6%)	6.960	0.031*
Overweight	300 (65.1%)	161 (34.9%)		
Obese	108 (69.7%)	47 (30.3%)		

*p < 0.05, **p < 0.01, ***p < 0.001

In addition, there was a significant positive correlation between age and respondents’ agreement that community pharmacists should provide weight management services (r_pb_ = 0.080, *n* = 730, *p* = .031), indicating that higher age was associated with greater acceptability of weight management services in community pharmacy.

Actual weight status and income of respondents did not show a significant relationship with acceptability of weight management services in community pharmacy.

## Discussion

The prevalence of obesity is increasing rapidly in developing countries such as Malaysia. Multiple public health interventions have been carried out worldwide in order to address the harmful effects linked to obesity. This study explored the Malaysian general public’s views in relation to weight management services provided by community pharmacists, an area which was relatively unknown. Community pharmacists are well-placed to assume responsibility in providing extended services in weight management, as shown in previous studies conducted in other countries such as USA [16], UK [20] and Australia [21]. Additionally, previous studies in Malaysia have documented the willingness of community pharmacists in providing extended, value-added services [18, 22, 23].

The weight reduction measures that were most strongly agreed or agreed by the respondents to help them achieve weight loss were exercise training and counselling by dieticians. The largest percentage of respondents also ranked dieticians as the preferred first line of consultation that could help them in weight reduction. These findings suggest that majority of respondents still favoured lifestyle interventions such as diet and physical activity in weight management. Conversely, only about a quarter of respondents ranked community pharmacists as their preferred first or second line of consultation on weight management. This finding is consistent with a previous study by Krska et al. (2010), which found that pharmacists were not favoured as sources of advice on weight management. Previous studies have also suggested that community pharmacy users were not willing to discuss healthy lifestyles with pharmacists, as they may have viewed pharmacists as medication experts only and not as experts on health and illness [24, 25]. This is supported by the current study, where less than half of the respondents perceived that community pharmacists could provide advice related to physical activity and weight management. Similar to previous studies [26], respondents also believed that weight management requires teamwork among various healthcare professionals; highlighting the importance of a multidisciplinary team approach that targets all lifestyle areas when developing pharmacy-based weight management services.

In order to provide weight management services or programs in community pharmacies, it is important for consumers to first perceive the availability of these services. In the present study, a large majority of the respondents were aware about services offered in the pharmacy related to measurement of health indicators such as blood pressure, blood glucose and blood cholesterol. Fewer respondents perceived the availability of services for measuring parameters more directly related to weight management though, such as body mass index, waist/hip circumference and body fat percentage. Respondents in this study also did not perceive pharmacists’ roles as a source of referral to other healthcare providers such as the doctors or dieticians. It is possible that some of the listed services were not available in the community pharmacies that the respondents had visited. However, another possible explanation is that respondents lacked awareness about the range of health services available through community pharmacy. Previous studies in other countries have also identified that lack of patient expectations is an important barrier in providing pharmacy-based weight management services to obese patients [27, 28]. Similar findings were concluded by a recent systematic review, which found that despite positive patient and public opinions about community pharmacy services in the UK, awareness about the services provided remains low [13]. More can be done to promote the roles of pharmacists in these areas to the general public who may benefit from weight management interventions.

Moreover, despite perceived availability on some weight management services and weight loss products in community pharmacies, it was important to note that only a small percentage of the respondents reported utilizing these services or purchasing these products. This was despite the majority of respondents in our study being categorized as overweight and obese based on their perceived image and actual BMI, and to whom weight management services would be targeted. This finding is supported by a study carried out by Weidmann et al. [28]. Given that community pharmacists frequently encounter people who may benefit from losing weight, it is important to identify the reasons for this low utilization and to develop strategies to reduce the gap for public in obtaining these services.

A positive finding was that the majority of respondents (64.4%) were in favour of community pharmacists providing weight management services. In line with previous studies, this support appeared to be higher among those who are female, with higher age and lower education, and having self-perceived body image as overweight or obese. A study by Fakih et al. (2014) has highlighted women pharmacy consumers’ positive attitudes towards receiving advice from their community pharmacist regarding weight management [29]. Additionally, the study by O’Neal et al. (2012) found that higher age and less education were associated with greater interest in pharmacist-delivered weight management services [16]. The study also found that most respondents who were willing to pay out of pocket for these services were overweight or obese. These findings may provide insight into targeting strategies for community pharmacy-based weight management services.

Based on findings from this study, factors that may influence the acceptability of weight management services in community pharmacy include perceived training for pharmacists, and the issues of payment for pharmacy services, privacy and wait time for services. This study found that 40% of respondents felt that community pharmacists are sufficiently trained to manage overweight and obese patients. This is comparable to an Australian study which found that about a third of pharmacy consumers thought that the pharmacist has the skills to offer a weight management service [26]. Pharmacists themselves have identified areas of training needs including physical assessment skills, consultation skills (motivational interviewing, goal setting) and evidence-based advice on weight loss products and drugs [30]. In addition, lack of time, lack of private consultation space and issues of payment/remuneration have been previously noted by both consumers and community pharmacists as important barriers in providing weight management services [26, 27]. A recent review have identified time pressure, excessive workload, inadequate training, a poor community pharmacy environment, and a lack of reimbursement, among other factors, as barriers in the delivery of public health services in community pharmacy [31].These barriers would thus need to be considered and addressed in developing and implementing these services in community pharmacies.

To the best of the authors’ knowledge, this study was the first in Malaysia to look at the general public’s views on weight management services provided in community pharmacies. This study has provided insight on future service development and scope on further research in this area. However, some study limitations need to be noted. Although a large sample size was obtained, the location of data collection was limited to Klang Valley, Malaysia, an urban area. Hence, this may affect the generalizability of the findings. A larger, national survey, including both urban and rural areas, is recommended to examine whether the findings from this study is representative of the Malaysian general population. Moreover, convenience sampling was used in this study and may result in a possibility of selection bias. Additionally, as the study utilized a self-administered questionnaire, there could be a possibility of social desirability bias, whereby the respondents could intentionally provide a positive response perceived to be favourable to the researcher.

## Conclusions

The findings of this study demonstrated that the majority of respondents were in support of community pharmacy-based weight management services; however only a low percentage reported utilizing these services. Factors influencing acceptability of services included payment, waiting time and the issue of privacy. With adequate training of community pharmacists and increased awareness of services among the public, community pharmacists could play a larger role in addressing the issue of obesity in Malaysia.
